# Explainable machine learning reveals diverse yield-determining factors among Thai rice farmer cohorts: Implications for targeted agricultural support

**DOI:** 10.1371/journal.pone.0349688

**Published:** 2026-06-15

**Authors:** Manusnan Suriyalaksh, Benjapon Prommawin, Pimkhuan Hannanta-anan, Tanee Sreewongchai, Parichart Promchote, Sutkhet Nakasathien

**Affiliations:** 1 Department of Agronomy, Faculty of Agriculture, Kasetsart University, Bangkok Thailand; 2 Faculty of Economics, Chiang Mai University, Chiang Mai Thailand; 3 Department of Biomedical Engineering, School of Engineering, King Mongkut’s Institute of Technology Ladkrabang, Bangkok Thailand; Science Hub Nepal, NEPAL

## Abstract

Rice yield prediction and optimization remain crucial challenges in Thailand’s agricultural sector. This study presents an explainable machine learning framework for predicting farm-level rice yields and identifying key factors affecting productivity. We collected comprehensive data from 1,722 smallholder farmers in central Thailand, encompassing 58 agronomic and economic variables. Four automated machine learning (AutoML) frameworks – AutoGluon, auto-sklearn, h2o, and mljar – were evaluated using 5-fold cross-validation, with AutoGluon achieving the best performance (root mean square error: 0.532 tonnes/hectare, mean absolute error: 0.372 tonnes/hectare, R²: 0.538). Using global SHapley Additive exPlanations (SHAP) analysis, we identified farmers’ social networks, rental costs during harvest, and total harvesting expenses as the most influential predictors of rice yields. Notably, stronger social network connectivity was associated with higher yields, suggesting that information sharing and collective knowledge within farming communities play a key role in improving productivity. Clustering analysis based on individual SHAP values revealed six distinct farmer cohorts, each characterized by unique patterns of feature importance. These cohort-specific insights demonstrate the potential of combining AutoML with explainability techniques to move beyond uniform agricultural recommendations towards precision support tailored to the specific needs of different farmer cohorts.

## Introduction

Rice (*Oryza sativa* L.) is a staple crop in Thailand, with the country ranking as the sixth-largest global producer in the 2022/2023 season [1]. Despite this prominence, Thailand’s average yield of 2.86 tonnes per hectare lags behind regional peers such as Vietnam (6.1 tonnes/hectare) and Indonesia (4.72 tonnes/hectare) [1]. This yield gap has been attributed to multiple factors, including the predominance of rainfed lowland production and limited adoption of advanced yield-enhancing innovations such as smart agriculture, which have shown to reduce production costs and improve resource efficiency [2–5]. Accurate yield prediction systems offer one pathway to address this challenge by enabling evidence-based resource management and farming practices [6–8].

Machine learning (ML) methods have emerged as a promising approach for agricultural yield prediction, offering advantages over traditional process-based and simulation models by capturing complex, non-linear interactions among environmental, genetic, and management factors [9–14]. However, conventional ML workflows require substantial data science expertise for algorithm selection, hyperparameter tuning, and feature engineering. Automated Machine Learning (AutoML) addresses this barrier by automating these steps — systematically evaluating multiple algorithms, optimising their parameters, and combining them into ensemble models [15]. This automation renders sophisticated predictive analytics accessible to domain experts who may lack programming or data science backgrounds. AutoML frameworks also incorporate methods for handling multicollinearity, high dimensionality, and variable redundancy without extensive manual pre-processing [16–17], with the resulting ensemble models often outperforming individual traditional algorithms [18–19]. These capabilities make AutoML particularly suited to agricultural data, where variables are numerous, interdependent, and often redundant.

Predictive accuracy alone, however, is insufficient for practical agricultural applications. Stakeholders require interpretable insights that translate predictions into actionable recommendations. Studies show that explainable machine learning approaches can reveal relationships between environmental factors and crop yields, providing valuable planning information [20–21]. Liu et al. identified critical yield prediction factors through hidden feature analysis [22], while Marcinkevičs and Vogt recommended SHAP due to its mathematical rigor and compatibility with ensemble models [23]. Among explainability approaches, SHAP (SHapley Additive exPlanations) offers a model-agnostic method for quantifying feature contributions at both global and local levels, with mathematical foundation and compatibility with ensemble models [23–24]. This interpretability is particularly important in Thailand, where rice farming practices vary significantly within small geographical areas, necessitating location-specific and farmer-specific insights [25–26].

Despite growing ML applications in agriculture globally [7–9,13,14], their implementation in Thai rice systems remains constrained by three research gaps. First, existing studies have focused on traditional algorithms such as artificial neural networks and random forests, without systematic comparison AutoML frameworks [27–29]. Second, prior efforts have relied on aggregated regional-scale data [27–29], neglecting farmer-level variability in management practices, soil conditions, and socioeconomic constraints—particularly relevant given that smallholders constitute 80% of Thailand’s rice producers [26]. Third, ML applications in Thai agriculture have prioritized predictive accuracy over interpretability [27–29], limiting their translation into actionable farmer recommendations.

This study addresses these research gaps through an integrated framework combining AutoML optimization with SHAP explanability analysis for farm-level rice yield prediction in Thailand. Specifically, we: 1) develop a comprehensive dataset from 1,722 smallholder farmers in central Thailand with farm characteristics, management practices, and socioeconomic factors; 2) compare four open-source AutoML frameworks (auto-sklearn, AutoGluon, h2o, and mljar) to identify optimal farm-level yield prediction approaches; and 3) apply SHAP analysis to reveal key yield-influencing factors across distinct farmer cohorts.

## Methodology

**Fig 1:**
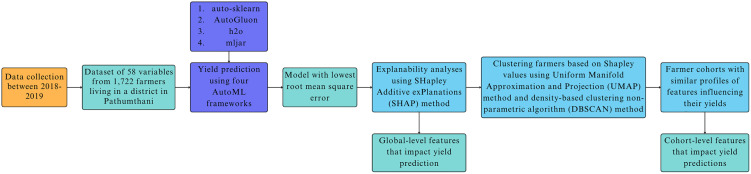
Explainable machine learning framework for rice yield prediction, comprising three sequential stages: data collection of 58 variables from participating farmers (detailed in Table 1), yield prediction using multiple machine learning models, and explainability analyses. Colour distinguishes study methods (orange, purple and blue) from results (green).

The overall study framework is illustrated in Fig 1. The methodology comprises three stages: data collection, yield prediction using AutoML, and explainability analysis using SHAP.

### Study design

#### Study area.

The study was conducted in a single district of Pathumthani province, Thailand, encompassing 41,363.2 hectares. Pathumthani is located within Thailand’s Central Plains region, which is characterized by relatively uniform climatic conditions, soil types, and irrigation infrastructure [30]. This geographical constraint was deliberately chosen to minimize confounding effects from environmental heterogeneity that could obscure relationships between management practices and yield outcomes [31]. By focusing on one district, we control for major environmental variables such as rainfall patterns, temperature regimes, and soil quality that vary substantially between regions and influence rice yields [31]. Although this constraint limits immediate generalizability, it creates a controlled environment for testing our integrated AutoML-SHAP methodology.

#### Variable selection.

The selection of predictor variables was guided by a theoretical framework that considered both agronomic principles and the practical realities of smallholder rice farming systems. We identified 58 variables across three main categories—administrative data, agronomic characteristics, and itemized farming expenses (Table 1).

**Table 1:** Data variable collected from farmers in Pathumthani province.

Administrative data	Agronomic characteristics	Farming expenses
Province - District - Subdistrict - Moo (village, smaller than subdistrict) - Farmer’s network (community of farmers sharing familiarity and agricultural knowledge)	- Yield - Total income - Expected harvest date - Planting date - Cultivation type - Rice variety - Seed age (days) - Plot size (hectare)	- labour cost for each of these processes: transportation, harvesting, spraying, pest management, weed management, water management, fertilizing, growing, seed preparation, soil preparation, others - rental cost for each of these processes: transportation, harvesting, spraying, pest management, weed management, water management, fertilizing, growing, seed preparation, soil preparation, others - material cost for each of these processes: transportation, harvesting, spraying, pest management, weed management, water management, fertilizing, growing, seed preparation, soil preparation, others - Total cost for each of these processes: transportation, harvesting, spraying, pest management, weed management, water management, fertilizing, growing, seed preparation, soil preparation, others - Total expense

Table 1 Variables collected from 1,722 farmers via a mobile application from September 2018 to August 2019. Labour cost refers to the cost of labour for specified processes; rental cost refers to the cost of renting equipment or services; material cost refers to the cost of materials used.

Our variable selection followed four key criteria: 1) demonstrated influence on rice yields in previous studies; 2) controllability by farmers, representing decision points during cultivation; 3) potential for targeted interventions; and 4) measurability within the constraints of our field data collection protocol. This approach ensured that our model included practically relevant variables while maintaining analytical rigor.

For administrative data, we included farmer’s network as another predictor variable due to the established importance of social learning and knowledge diffusion in agricultural communities. Aker (2011) documents how farmer-to-farmer knowledge networks function as critical pathways for information exchange regarding optimal practices, particularly in contexts where formal extension services are limited [32].

Agronomic characteristics were selected based on their documented impact on yield formation processes. Cultivation type and rice variety were incorporated as factors accounting for genetic and management-related yield variations, with meta-analyses showing these factors can explain 20–35% of yield variability in tropical rice systems [33–35]. Seed age at planting was included based on evidence that it affects early crop establishment, tillering capacity, and subsequent yields [36]. Plot size was incorporated to capture scale-related efficiencies in resource allocation and mechanization potential [37–38].

Farming expense variables were categorized by agricultural process (transportation, harvesting, pest management, etc.) following the management intensity framework proposed by Lobell et al. [39]. These expense categories serve as quantifiable proxies for management intensity and resource allocation patterns, with previous studies demonstrating correlations between input expenditures and yield outcomes in smallholder systems [40–41].

This approach to variable selection ensured that our model captured relevant factors influencing rice productivity in smallholder farming systems, while excluding variables that would have limited practical application for improving farmer outcomes.

### Data collection process

The study employed a self-reporting methodology, wherein farmers input data through questionnaires via a mobile application installed on their personal devices. Prior to participation, farmers were informed of the study’s purpose and data usage through village headmen, who facilitated and witnessed the registration process. Consent was obtained verbally and through the voluntary act of registering for and downloading the mobile application; by completing registration, farmers acknowledged their understanding of the study’s purpose and agreed to participate. All data were anonymized before access and analysis. This study was exempt from requiring ethics approval according to the ethics regulations for research involving human subjects set out by Kasetsart University, which conform to the National Policy and Guidelines for Human Research 2015 enacted by the National Research Council of Thailand. The Institutional Review Board (IRB) regulations of Kasetsart University granted the exception because the data were observational and self-reported through questionnaires, and that participation was entirely voluntary with no intervention or experimental manipulation involved.

The research team implemented a network-based strategy, leveraging existing social networks among farmers. A farmer’s network was defined as a community of farmers with established relationships, regardless of geographical proximity. Local follow-up sessions, organized by farmer leaders within these networks, provided ongoing support and encouraged consistent participation. This approach was designed to enhance participation rates and ensure data reliability through existing social structures.

Data were collected from 1,722 smallholder farmers during a complete agricultural cycle from September 1, 2018, to August 31, 2019. The collected variables encompassed 58 features spanning farm characteristics, management practices, and socioeconomic factors (Table 1). Cultivation type refers to the method of cultivation used (e.g., direct seeding, transplanting). Seed age refers to the number of days at the time of planting, which can affect early crop establishment and subsequent yields. Seven entries with zero yields were excluded from further analysis, resulting in a final sample of 1,715 farmers.

### Comparative yield prediction accuracy among AutoML frameworks

We selected four open-sourced AutoML frameworks—auto-sklearn, AutoGluon, h2o, and mljar—each representing different approaches to automated machine learning. The selection criteria include: 1) open-source accessibility with active maintenance, 2) automated data preprocessing capabilities, 3) algorithm diversity with minimum four base models including neural networks, and 4) ensemble methods combining multiple algorithms to enhance predictive performance. These criteria ensure framework accessibility for agricultural practitioners while addressing limitations of previous Thai rice yield prediction studies that employed only one to two machine learning algorithms [27–29]. The inclusion of neural networks and ensemble methods was designed to capture complex non-linear relationships within agricultural datasets [13], while the accessibility and data preprocessing requirements promote adoption by practitioners with varying technical expertise.

The selected frameworks employ two common model combination strategies, which we distinguish here. Ensemble learning combines predictions from multiple independently trained models — typically through averaging or voting—to improve predictive performance [42–43]. Stacking (or stacked generalisation) is a specific form of ensemble learning in which predictions from base models are used as input features for one or more subsequent meta-models, which learn the optimal way to combine them [43–44]. Table 2 summarises the key characteristics of these four frameworks, highlighting their optimisation approaches, ensemble methods, and interpretability features. While all frameworks share the common goal of automating the machine learning workflow, they employ distinct strategies for model selection, hyperparameter tuning, and model combination, which may result in different performance outcomes for our specific yield prediction task.

**Table 2:** Key characteristics of the four AutoML frameworks evaluated in this study.

Feature	Auto-sklearn	AutoGluon	h2o	mljar
**Optimization approach**	Bayesian optimization (SMAC)	Hyperparameter optimization with multi-layer stacking	Random grid search and genetic algorithm	Random search and genetic algorithm
**Meta-learning**	Yes	No	No	No
**Ensemble method**	Ensemble selection	Multi-layer stacking	Stacked ensembles	Model stacking
**Time allocation**	Fixed per model	Adaptive	Dynamic based on performance	Fixed total budget
**Preprocessing**	Automated feature preprocessing	Automated with specialized preprocessors	Automated with basic options	Extensive EDA and feature engineering
**Interpretability features**	Limited	Feature importance	Variable importance, partial dependence	Comprehensive explanations and visualizations
**Unique strength**	Meta-learning for warm start	Multi-layer stacking approach	Distributed computing for large datasets	Detailed reporting and explanations
**Implementation version**	0.14.6	1.0.0	3.4	0.10.0

The following subsections provide additional detail on each framework’s approach and our specific implementation parameters. Table 3 lists the base machine learning algorithms included in the ensemble and stacking processes for each framework.

**Table 3:** Base machine learning algorithms included for the four frameworks.

AutoML framework	Base algorithms included in the experiment
Auto-sklearn	Random Forest, Extra Trees, Gradient Boosting, Multilayer perceptron, Passive Aggressive, Stochastic Gradient Descent
AutoGluon	Random Forest, Extra Trees, XGBoost, LightGBM, CatBoost, Linear Models, Neural Networks (torch, fast.ai)
h2o	Distributed Random Forest, XGBoost, GBM, Extremely Randomized Trees, GLM (with regularization), DeepLearning
mljar	Random Forest, Extra Trees, XGBoost, LightGBM, CatBoost, Decision Tree, Neural Network, Nearest Neighbors

### Auto-sklearn

Auto-sklearn employs a meta-learning approach to warm-start the optimization process, leveraging Bayesian optimization (specifically Sequential Model-based Algorithm Configuration) to efficiently navigate the configuration space of machine learning algorithms and hyperparameters [42]. It constructs ensembles from the best-performing models discovered during optimization by combining their predictions through weighted averaging. For our implementation, auto-sklearn (version 0.14.6) was configured to optimize for mean squared error with 5-fold cross-validation and a time limit of 600 seconds per run, with 14,400 seconds allocated for the complete task.

### AutoGluon

AutoGluon distinguishes itself through a multi-layer stacking approach [43]. It first trains diverse base algorithms independently, then combines their predictions using additional models in subsequent layers — a process repeated across multiple stacking levels. Its strength lies in this stacking strategy and adaptive time-budgeting. We implemented AutoGluon (version 1.0.0) with the “good_quality” preset parameter and included XGBoost, Gradient Boosting Machines, CatBoost, Extra Trees, Random Forest, Neural Networks, and FastAI models. We enabled auto-stacking and implemented 5-fold validation for consistency with other frameworks.

### h2o

h2o AutoML employs a combination of random grid search and a modified genetic algorithm for hyperparameter tuning, with intelligent resource allocation that dynamically adjusts time spent on different algorithms based on their performance [44]. It generates a leaderboard of models, including both individual algorithms and stacked ensembles— the latter using a meta-learner trained on the predictions of base models. Our implementation (version 3.4) limited the maximum models to 60 and utilized 5-fold cross-validation for consistency across frameworks.

### mljar

mljar emphasizes transparency, interpretability, and user-friendly reporting, employing a multi-step process that includes extensive exploratory data analysis and feature engineering [45]. It utilizes a combination of random search and a genetic algorithm for hyperparameter optimization. mljar supports both ensemble averaging and stacking, which can be enabled independently or in combination. Our implementation set the mode to “Optuna” with a 14,400-second time budget, explanation level 2 for model interpretability features, and 5-fold cross-validation with both ensemble training and model stacking enabled.

### Rice yield prediction implementation

From the four-selected frameworks, we predicted rice yields per hectare using 54 input features (see S2 File). These features consist of the variables listed in Table 1 excluding: 1) yield data (target variable), 2) total income (high correlation with yields), and 3) province and district identifiers (uniform across all farmers).

### Validation

To evaluate and compare the performance among different methods, we employed three widely-used statistical metrics: root mean square error (RMSE), mean absolute error (MAE), and coefficient of determination (R²). Each metric offers distinct advantages and limitations in assessing model performance. RMSE has been widely adopted in agricultural modelling studies [46] due to its sensitivity to large errors, which is particularly relevant in crop yield prediction where significant deviations could have substantial practical implications. However, RMSE’s quadratic scoring rule makes it more susceptible to outliers compared to MAE. MAE, while more robust to outliers, provides a more intuitive interpretation of error magnitude in the same unit as the response variable. Nevertheless, both RMSE and MAE are scale-dependent, limiting their utility in cross-dataset comparisons [47]. R², meanwhile, offers scale-independence and intuitive interpretation of the proportion of variance explained by the model, but it can be misleading when models are biased or when the relationship between variables is nonlinear [48–49]. In our analysis, we prioritized RMSE as the primary metric while using MAE and R² as complementary measures to provide a more comprehensive assessment of model performance [47–48]. To ensure robust evaluation, we implemented k-fold cross-validation with k = 5, a widely adopted configuration that balances bias and variance in performance estimation [50]. The dataset was divided into five subsets, with four used for training and one for validation, rotating across all five folds.

### Explainability analyses

As identified in the introduction, ML applications in Thai agriculture have prioritised predictive accuracy over interpretability [27–29], limiting their practical value for understanding yield variability among smallholder farmers. To address this gap, we employed SHAP values to interpret the relationships between input features and predicted rice yields [51]. SHAP values, based on cooperative game theory, provide a unified approach to explaining the output of any machine learning model by quantifying the contribution of each feature to individual predictions [52]. This enables both global and local interpretability, both of which are essential for generating farmer-specific insights from our AutoML-derived yield prediction model.

### SHAP methodology

The SHAP methodology decomposes an individual prediction into the sum of feature contributions by calculating how each feature value shifts the prediction away from the expected average prediction for the dataset. Mathematically, for a given prediction f(x), the SHAP value ϕᵢ for feature i represents its contribution to the difference between the actual prediction and the base value (average prediction across the dataset):


$$\begin{array}{c} f(x)\;=\;E[f(X)]\;+\;\Sigma_{i}\phi_{i}\; \end{array}$$

Where E[f(X)] is the average prediction across all instances, and ϕᵢ values sum to the difference between the individual prediction and this average [52].

### SHAP implementation

We implemented SHAP analysis using the SHAP Python package (version 0.45.0) on the best-performing model identified in the framework comparison. We chose Kernel explainer for its ability to explain any model including ensembled models and complex feature processing [53]. The ability to handle any models of the Kernel explainer comes with high computation cost as it simulates each feature being missing by replacing the respective feature value with a random value from the dataset. Therefore, we applied stratified sampling for 250 samples from the dataset to calculate the SHAP values (refer to S4 File for implementation details). Lundberg and Lee demonstrated that sampling-based approximations of Shapley values can provide reliable feature importance estimates while significantly reducing computational overhead [51].

Our SHAP analysis proceeded in three stages. First, we conducted a global analysis by averaging the absolute SHAP values across all farmers to identify the most influential features overall. Second, we generated SHAP summary and dependence plots to visualize feature effects on predictions. Finally, we performed farmer clustering based on their individual SHAP value profiles across all 54 features, grouping farmers with similar patterns of feature importance. We applied Uniform Manifold Approximation and Projection (UMAP) for dimensionality reduction of the 54-dimensional SHAP vectors [54] and Density-Based Spatial Clustering of Applications with Noise (DBSCAN) for clustering [55]. UMAP parameters were optimized by testing neighbor values from 10 to 40; values exceeding 22 caused cluster convergence to a single group, while 15 neighbors provided optimal cluster separation. The final number of clusters was determined using the silhouette coefficient.

This multi-level SHAP analysis provided insights at both aggregate and cohort-specific levels, enabling us to identify not only which features most influenced yield predictions overall but also how these influences varied across different farmer cohorts. By connecting model predictions to interpretable feature impacts, SHAP analysis transformed complex model outputs into actionable insights.

## Results

### Data characteristics

**Fig 2:**
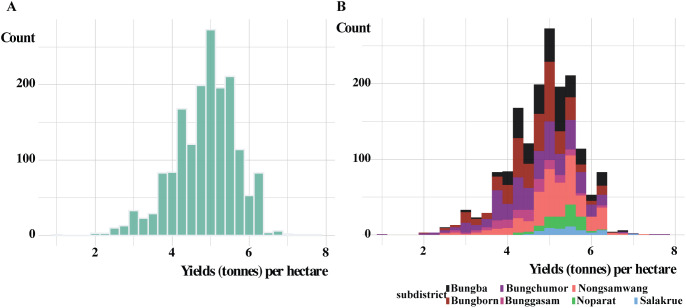
Distribution of rice yields in the study population. **A)** Frequency distribution of rice yields (tonnes/hectare). **B)** Frequency distribution stratified by seven sub-districts. Yield variability persists regardless of geographical location.

We assessed yield variability across farmers by analyzing the distribution of yields per hectare (calculation method in S6 File) using histogram visualization. Fig 2A shows substantial yield variation among farmers despite all plots being located within the same district. This variability could potentially be attributed to geographical differences between sub-districts. Fig 2B addresses this by showing the distribution by sub-district, revealing that farmers within the same sub-district also exhibit wide yield variation. Together, these observations indicate that yield variability persists regardless of geographical location, suggesting complex relationships among influencing variables that extend beyond environmental conditions. This justifies the application of machine learning to identify these underlying factors.

### Comparative yield prediction accuracy

Table 4 presents the best score from each of the selected AutoML frameworks (AutoGluon, auto-sklearn, H2O, and MLJAR); and Table 5 summarizes the performance range for each framework. The majority of the models (77.5%) employ ensemble methods. AutoGluon framework produced the best prediction model with RMSE of 0.532 tonnes/hectares, MAE of 0.372 tonnes/hectares, and R^2^ of 0.538 (lowest RMSE and MAE; and highest R^2^). The best model is based on an ensemble method from eight different base algorithms namely XGBoost, LightGBM, CatBoost, Neural network from fast.ai, Pytorch neural network, Linear model, Random Forest, and Extra tree model. The result establishes that the performance of the ensembling technique, which has not been shown in the context of rice yield prediction in Thailand, is superior to running single base algorithm predictions.

**Table 4:** Performance comparison of top models from each AutoML framework.

Framework	Best RMSE	MAE	R²	Base algorithms in best model
AutoGluon	0.532	0.372	0.538	XGBoost, LightGBM, CatBoost, FastAI, Neural Network, Linear Regression, Random Forest, Extra Trees
mljar	0.614	0.380	0.509	CatBoost, LightGBM, XGBoost, Neural Network, Random Forest, Extra Trees
auto-sklearn	0.634	0.392	0.479	Extra Trees
h2o	0.650	0.414	0.447	Distributed Random Forest, Extremely Randomized Trees, Generalized Linear Model with regularization, XGBoost, H2O Gradient Boosting Machine, DeepLearning

**Table 5:** Performance range summary by framework.

Framework	RMSE range	Performance variation within framework
AutoGluon	0.532-0.555	Higher diversity in algorithms improved performance (4.3% RMSE reduction)
mljar	0.614-0.625	Multiple algorithms outperformed single algorithm models (1.8% RMSE reduction)
auto-sklearn	0.634-0.642	Limited algorithm diversity with consistent performance (1.3% range)
h2o	0.650-0.677	Ensemble models outperformed single models (4.1% RMSE reduction)

* Performance variation calculated as the percentage difference between the best and worst performing models within each framework.

Overall, models from AutoGluon achieves superior result to the other frameworks. The framework ensembles multiple base algorithms right away in their optimization approach, rather than first optimizing for hyperparameters (details in S2 File). Note that all frameworks have multiple machine learning base algorithms enabled (Table 3), but depending on their optimization approach, they execute ensembling differently. This could offer an explanation for its superior performance given limited time to optimize for best results.

**Fig 3:**
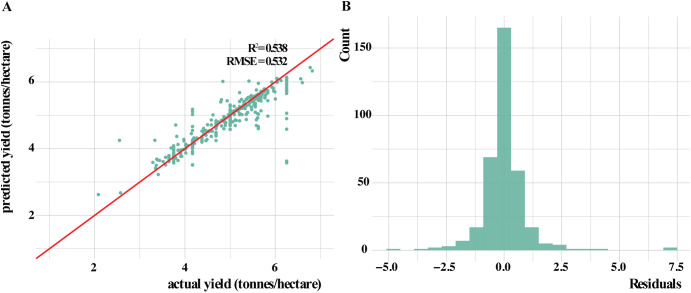
Performance evaluation of the best-performing AutoML model. A) Predicted versus actual rice yields (tonnes/hectare); the red line indicates y = x. B) Distribution of residuals from test dataset predictions.

Fig 3 illustrates the performance of the best-performing model — AutoGluon’s ensemble (Table 4) — by comparing predicted yields with actual yields and displaying the residual distribution. 74.65% of the test set residuals fell between −0.6 and 0.6 tonnes/hectares, consistent with the model’s RMSE of 0.532.

### Global-level explainability analyses of the best performing model using SHapley Additive exPlanations (SHAP) method

**Fig 4:**
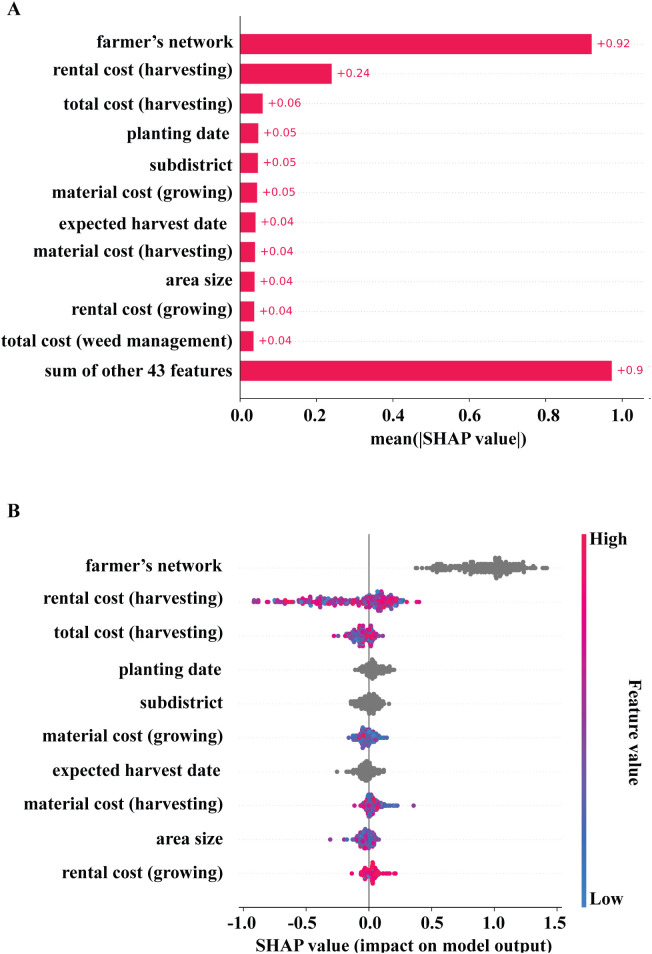
SHAP feature importance analysis for rice yield prediction. A) Global feature importance ranked by mean absolute SHAP values across all predictions. B) SHAP summary plot for the top 10 features. Each point represents an individual farmer; the x-axis indicates the direction and magnitude of feature impact on predicted yield. For numerical features, colour ranges from low (blue) to high (red) values; grey indicates categorical features. Both panels show the same feature set.

We conducted SHAP analysis to quantify feature importance in our best-performing model. The global feature importance analysis (Fig 4A) ranks features by mean absolute SHAP values across all predictions, representing each feature’s overall impact on rice yield predictions [52].

Farmers’ social networks emerged as the strongest predictor, exhibiting the highest mean absolute SHAP value. These networks facilitate information exchange, resource sharing, equipment arrangements, and collective input purchasing within farming communities, reflecting the critical role of social learning and knowledge diffusion in agricultural productivity. The second and third most influential features were rental costs during harvest and total cost during harvest, respectively, though their impact was notably lower. Higher rental costs during harvesting typically indicate access to efficient machinery or timely services that minimize grain losses during this critical phase. All remaining variables showed markedly lower SHAP values with minimal individual contributions to model predictions. These results highlight the importance of social factor, while suggesting that other variables may primarily serve as fine-tuning parameters rather than primary yield determinants.

To examine both the magnitude and directionality of feature effects, Fig 4B presents individual SHAP values across all observations. Unlike the global importance plot (Fig 4A) showing absolute values, the summary plot displays actual SHAP values (including positive and negative contributions), revealing how specific input features influence predictions for individual farmers and providing detailed insights into the model’s decision-making process. The farmers’ social network consistently demonstrated positive SHAP values across all instances, indicating its robust positive association with predicted yields. In contrast, rental costs exhibited a bimodal distribution of SHAP values, suggesting heterogeneous relationships with yield predictions across different farmers. While some instances showed positive SHAP values, others displayed negative values with notably larger magnitudes. This bimodal distribution indicates that rental costs’ relationship with predicted yields varies across different farming contexts, potentially reflecting diverse operational strategies, equipment access patterns, or cost-efficiency trade-offs among farmers in our sample. Nevertheless, the bimodal distribution could also reflect other phenomena including threshold effects where rental costs become beneficial only above or below certain levels, or interaction effects with other variables. The SHAP values’ heterogeneity across observations suggests that the model captured complex, non-linear relationships between predictive features and rice yields, rather than simple uniform effects. Total cost during harvest shows a similar trend to the rental cost but with lower magnitude effects.

**Fig 5:**
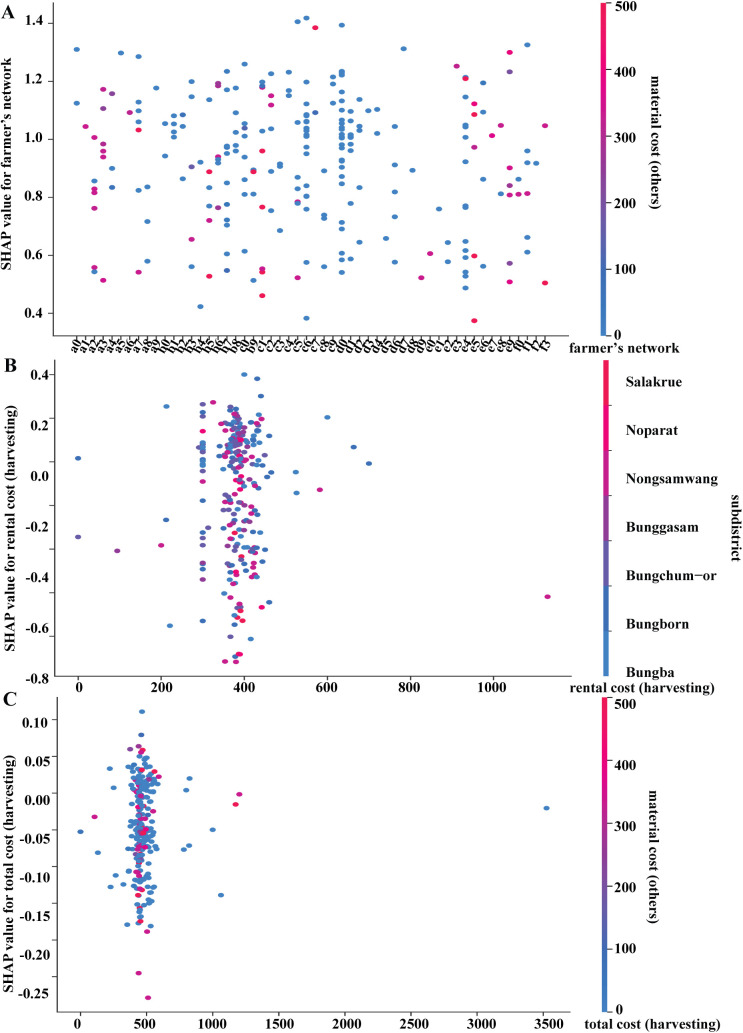
SHAP dependence plot for the three most influential features. A) farmer’s network, B) rental cost during harvesting, and C) total harvesting cost. Each point represents one observation; the x-axis shows the feature value and the y-axis its SHAP contribution to predicted yield. Colour gradients indicate the strongest interacting feature. For panels A and C, material costs exclude transportation, harvesting, spraying, pest management, weed management, water management, fertilising, growing, seed preparation, and soil preparation.

Next, we examined SHAP dependence plots for the three highest-importance features to explore their relationships with predicted yields and potential interactions (Fig 5). The farmers’ network dependence plot (Fig 5A) revealed distinct patterns where high material costs concentrated within specific network clusters rather than being uniformly distributed across all networks. This clustering suggests that certain farmer networks may facilitate access to higher-cost inputs or employ more intensive production strategies, though our observational data precludes causal inference. In contrast, the dependence plots for rental costs by sub-district location (Fig 5B) and harvesting costs by other material costs (Fig 5C) showed no clear interaction patterns, with SHAP values remaining relatively consistent across feature combinations. These visualizations demonstrate how network membership interacts with input decisions in our study population, while other key factors operate more independently in determining rice yields.

### Clustering farmers based on individual SHAP values

**Fig 6:**
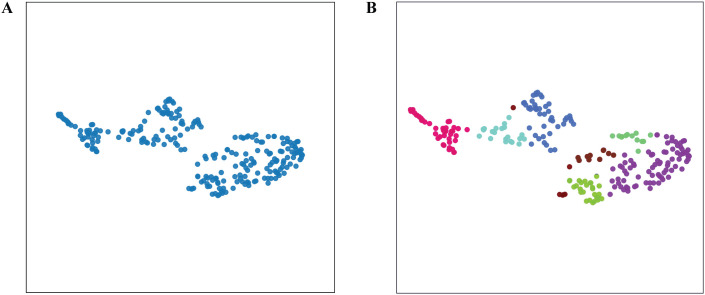
Two-dimensional UMAP embedding of SHAP values from 54 features for rice yield prediction. Each point represents an individual farmer, with spatial proximity indicating similar feature importance patterns. A) UMAP embedding of farmer SHAP profiles. B) DBSCAN cluster analysis of the embedded space; colours denote distinct clusters and dark purple points represent unclustered observations.

Based on the varying effects of features observed across individual farmers (Figs 4B and 5), we identified distinct farmer subgroups characterized by similar patterns of feature importance. UMAP dimensionality reduction of individual SHAP values revealed separable farmer clusters (Fig 6A), which were subsequently classified into six distinct groups using DBSCAN clustering (Fig 6B). This clustering analysis demonstrates that farmers in our sample operate under different yield-determining paradigms, with each cluster exhibiting unique combinations of influential factors affecting their rice productivity.

**Fig 7:**
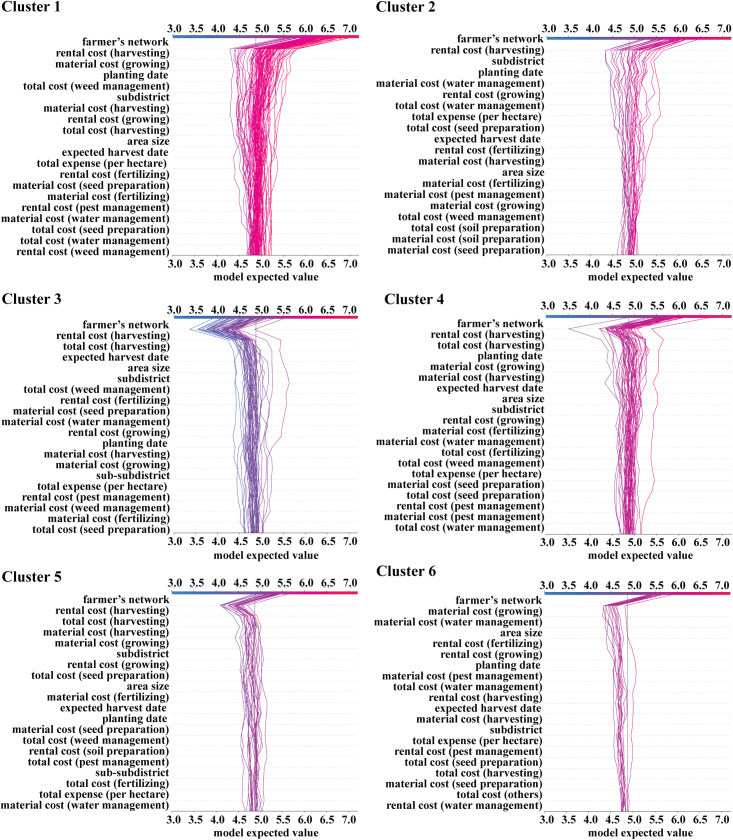
SHAP decision plots for rice yield prediction across DBSCAN-identified clusters. Each panel represents a distinct cluster; each line traces an individual farmer’s prediction pathway from the model’s expected value (bottom x-axis) to the final predicted yield (top x-axis, ranging from 3.0 to 7.0 tonnes/hectare). Features are ordered by overall importance along the y-axis.

To further examine the clustering result, we generated decision plots for each identified cluster (Fig 7). Decision plots provide a detailed visualization of how the model arrives at each prediction, tracking the cumulative impact of features from the expected value to the final prediction. The plot traces how each feature sequentially shifts the prediction from the expected value (the model’s average prediction at the bottom) to the final predicted value (at the top), with the slope indicating the magnitude and direction of each feature’s contribution. Features are ordered according to their SHAP value magnitude.

Within each identified cluster, we observed consistent trajectories in these decision pathways, indicating similar patterns in how features accumulated to form predictions. Between clusters, however, these trajectories showed distinct variations in both the ordering and magnitude of feature contributions. Fig 7, therefore, reveals the heterogeneity in feature importance across different farmer groups, highlighting how features such as farmer’s network, rental costs, and material costs vary in their influence on rice yield predictions among distinct farmer clusters. For instance, Cluster 1 and Cluster 4 (Fig 7) represent two cohorts where total cost during harvest contributes differently to model prediction. For Cluster 1, total cost during harvest ranks 9^th^ based on feature importance for the cohort, whereas in Cluster 4 it ranks 3^rd^, implying that total cost during harvest has higher impact to farmers in Cluster 4 than those in Cluster 1. This variation in feature importance ordering across clusters suggests that the identified groupings represent meaningful distinctions between each farmer cluster.

**Fig 8:**
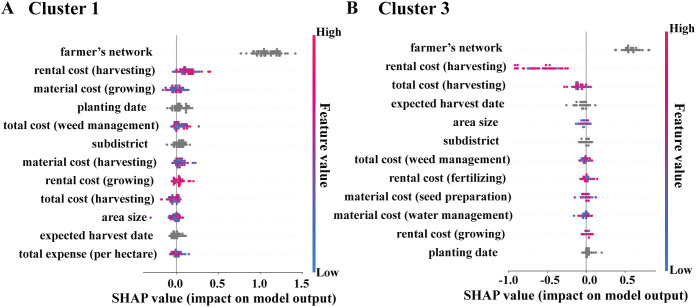
SHAP summary plot comparing between Cluster 1 and Cluster 3 for the top 10 contributing features to rice yield prediction. Each point represents an individual farmer; the x-axis indicates the direction and magnitude of feature impact on predicted yield. For numerical features, colour ranges from low (blue) to high (red) values; grey indicates categorical features. Features are ordered by overall importance.

Fig 7 revealed distinct ordering patterns in feature importance across clusters, with notable differences in predicted yields relative to the predicted mean. Cluster 1 (Fig 7) was characterized by predominantly above-average yield predictions. In contrast, Cluster 3 (Fig 7) showed consistently below-average yield predictions, with a different pattern of feature contributions compared to Cluster 1. While both clusters shared the importance of social networks and harvest rental costs, Cluster 1’s predictions were additionally influenced by growing material costs, weed management expenses, and planting date timing (Fig 8A). These three features showed substantially lower SHAP values in Cluster 3’s decision pathways (Fig 8B), suggesting that they are key differentiating factors between high- and low-yield farmer groups.

## Discussion

### Model performance and key findings

The AutoGluon framework produced the most accurate model for predicting rice yields among 1,722 smallholder farmers in Thailand, achieving RMSE of 0.532 tonnes/hectare. SHAP analysis identified farmers’ social networks, harvest rental costs, and total harvest costs as the most influential features, and clustering of individual SHAP values revealed six distinct farmer cohorts with different patterns of feature importance.

The model performance demonstrates reasonable accuracy when compared to recent studies in similar contexts, though most studies predicted yields at the aggregated country level. For instance, Jabjone reported RMSE values of 9.94 to 138.87 kg/rai, equivalent to 0.062 to 0.868 tonnes/hectare [27]. Another study employing a similar nature of input data reports RMSE of 0.752 tonnes/hectare using the widely-used WOFOST model in Central Thailand [56], placing our model’s performance within the established range of accuracy in the literature. Our results compare well with studies from other agricultural systems: Cao et al. [9] achieved an RMSE of 1.05 tonnes/hectare for farm-level wheat prediction in China, while Shahhosseini et al. [13] reported RMSE values of 0.84–1.29 tonnes/hectare for country-level corn prediction in the United States.

A key advantage of the AutoML approach is the systematic comparison of multiple algorithms and ensemble strategies, which in our case identified an ensemble model that outperformed any individual algorithm. This contrasts with previous Thai rice yield studies that employed only one or two algorithms without systematic optimisation [27–29].

### Integration of AutoML and SHAP for agricultural systems analysis

While AutoML and SHAP have been applied independently in agricultural research, their integration into an end-to-end workflow for farm-level yield prediction remains limited. Khaki et al. [57] applied a guided backpropagation method to interpret a CNN-RNN model for corn and soybean yield prediction in the United States, and Minamikawa et al. [58] used gradient-weighted class activation to explain predictions of fruit quality traits in Japan. More recently, several studies have combined SHAP with ensemble models in agricultural contexts: Yenkikar et al. [59] integrated SHAP and LIME with a hybrid ML model for rice yield prediction in India; Mohan et al. [60] combined SHAP and LIME with ensemble regressors to assess climate change impacts on crop yields; and Elbeltagi et al. [61] applied SHAP, LIME, and Sobol sensitivity analysis alongside tree-based models for soybean crop coefficient estimation in Egypt. These studies demonstrate the growing adoption of explainability techniques in agricultural prediction. However, in each case, explainability was used to interpret overall model behaviour and identify influential features globally, without extending the analysis to distinguish typologies among individual production units (e.g., farmers or fields) based on their feature importance patterns. Our study builds on these works by clustering individual SHAP values to derive data-driven farmer cohorts, each characterised by different patterns of yield-influencing factors. This additional step enables cohort-specific insights that would not emerge from either predictive modelling or global explainability analysis alone, addressing the growing need for interpretable AI in agricultural research [62–63].

### Implications for understanding agricultural systems

Our results demonstrate that farmers operating under similar biophysical conditions exhibit different yield outcomes, driven by distinct combinations of socioeconomic and management factors. This finding is consistent with observations in other smallholder rice systems. Kwesiga [64] demonstrated large exploitable yield gaps in Tanzanian floodplain rice systems, attributing them primarily to differences in crop management practices across field environments. Similarly, Niang et al. [65] analysed 1,305 farmer fields across 11 West African countries and identified management factors — including nitrogen application, weeding frequency, and seed choice — as key determinants of yield variation alongside climatic and soil conditions. In both cases, management-related factors explained substantial yield differences even within similar production environments.

The differences in feature importance between Cluster 3 (low-yield) and Cluster 1 (high-yield) provide insights into the mechanisms underlying these productivity differences. While both clusters shared the influence of social networks and harvest rental costs, high-yield farmers were additionally differentiated by growing material costs, weed management expenditure, and planting date timing. These cohort-specific patterns suggest that uniform approaches may obscure farmer heterogeneity, consistent with the principles of precision agriculture and data-driven farm management outlined by Wolfert et al. [66].

The prominence of farmer networks as a yield-influencing factor aligns with Aker’s [32] analysis of information diffusion in agricultural systems. Aker [32] reviewed the potential mechanisms through which information and communication technologies could facilitate agricultural technology adoption in developing countries, highlighting the role of social networks and extension services in mediating information flow to farmers. This suggests that social connectivity may function as a pathway through which agricultural knowledge and practices are disseminated, providing a plausible mechanism for the network effects observed in our study.

Taken together, these findings suggest that agricultural programmes and extension services may benefit from farmer segmentation approaches rather than uniform recommendations, an approach increasingly advocated by the FAO [67] and CGIAR [68] for effective technology adoption and policy implementation. Similar cohort-based approaches have shown promise in smallholder systems in Vietnam [69], suggesting potential transferability of this methodology to other Southeast Asian contexts where comparable heterogeneity exists.

### Limitations and future research directions

Several limitations should be acknowledged. First, our study focuses on a single province in Thailand, which may limit the generalisability of specific findings to other regions with different agro-ecological conditions, cropping systems, or socioeconomic contexts. The relative importance of features such as social networks or harvest costs may differ in regions with different labour markets or mechanisation levels. Validating this framework across multiple provinces or countries would help distinguish context-specific from generalisable patterns.

Second, the cross-sectional nature of our data, collected over a single agricultural cycle (2018–2019), prevents analysis of temporal dynamics in farmer typologies and yield patterns. Farmer cohort membership may shift across seasons due to changes in management practices, market conditions, or climate variability. Longitudinal studies tracking the same farmers over multiple growing seasons would be needed to assess the temporal stability of the identified typologies.

Third, while our framework identifies important yield-influencing factors through SHAP analysis, the associations identified are correlational rather than causal. For example, the prominence of social networks as a yield-influencing factor does not establish whether network participation directly improves yields or whether higher-performing farmers are more likely to participate in networks. Establishing causal relationships would require complementary experimental or quasi-experimental research designs.

Fourth, the self-reported nature of the data collected via mobile application may introduce measurement error or reporting bias, particularly for cost-related variables where farmers may estimate rather than record exact figures. Future implementations could incorporate validation mechanisms such as cross-referencing with administrative records or satellite-derived crop metrics.

Future research should examine whether the farmer cohorts identified in this study remain stable across multiple growing seasons, as farmers may shift between groups over time in response to changing practices, market conditions, or climate. Applying this framework to different crops and regions would also help determine which findings are specific to this context and which are more broadly applicable. Additionally, pairing this analytical approach with field-based experiments would help establish whether the influential factors identified through SHAP analysis have a direct causal effect on yield outcomes.

## Conclusion

This study demonstrated the effectiveness of combining AutoML with SHAP analysis for predicting rice yields and identifying key influencing factors in Thai smallholder farming systems. The AutoGluon framework achieved an RMSE of 0.532 tonnes/hectare, providing predictions comparable to existing yield models in similar contexts and interpretable insights into the determinants of rice productivity.

SHAP analysis identified farmers’ social networks, harvest rental costs, and total harvest costs as the most influential features. The prominence of social networks suggests that they function as a form of information capital — facilitating the exchange of agricultural knowledge, practices, and resources that collectively improve productivity. Clustering of individual SHAP values revealed six distinct farmer cohorts, each characterised by different patterns of feature importance. These findings suggest that yield variability among smallholders is driven by diverse combinations of management and socioeconomic factors rather than a uniform set of constraints. This challenges traditional one-size-fits-all agricultural recommendations and supports a shift towards precision policy approaches tailored to the specific needs and constraints of different farmer cohorts.

Future work should examine the temporal stability of these farmer typologies across multiple growing seasons, as cohort membership may evolve over time in response to changing practices and conditions. Validating the transferability of this framework to other crop systems and geographical contexts would further establish its broader applicability.

## Supporting information

S1 Fig The SHAP global feature importance distribution of the top-performing model when removing total cost variables. (TIF)

S1 File Sensitivity analysis of variable redundancy in cost categories. (DOCX)

S2 File Automated machine learning frameworks. (DOCX)

S3 File Evaluation metrics. (DOCX)

S4 File Model explainability using SHapley Additive exPlanations (SHAP) values. (DOCX)

S5 File Clustering farmers based on SHAP values using UMAP and DBSCAN. (DOCX)

S6 File Data preparation. (DOCX)
