# Supplementary figures and images for: Explainable machine learning reveals diverse yield-determining factors among Thai rice farmer cohorts: Implications for targeted agricultural support
Source: PLoS One. 2026 Jun 15;21(6):e0349688. doi: 10.1371/journal.pone.0349688 (PMC13268196; doi:10.1371/journal.pone.0349688)

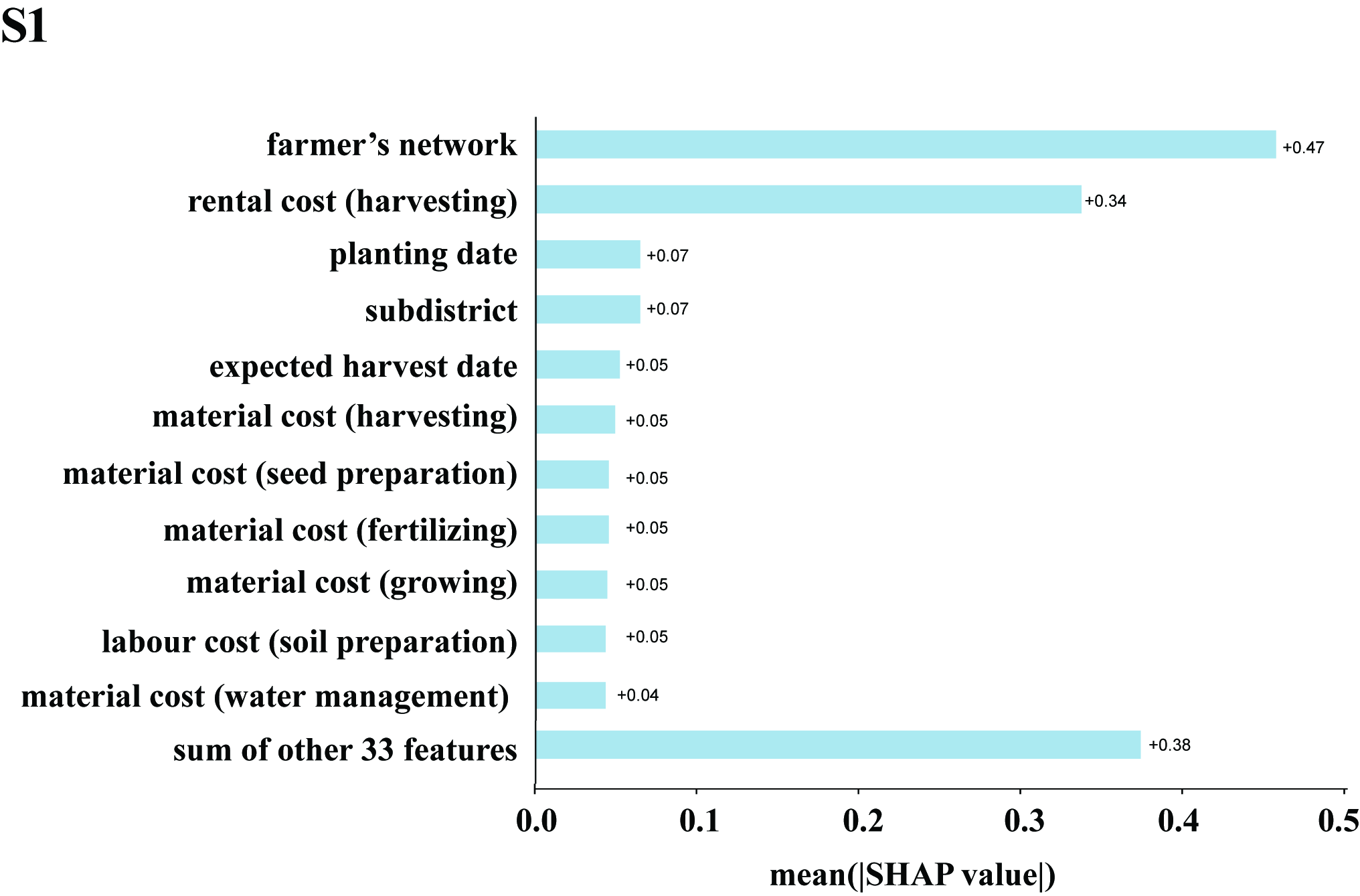

Supplement: S1 Fig — (TIF) [file pone.0349688.s001.tif]
