# Supplementary material for: Explainable machine learning reveals diverse yield-determining factors among Thai rice farmer cohorts: Implications for targeted agricultural support
Source: PLoS One. 2026 Jun 15;21(6):e0349688. doi: 10.1371/journal.pone.0349688 (PMC13268196; doi:10.1371/journal.pone.0349688)
Supplement: S1 File — (DOCX) [file pone.0349688.s002.docx]

**Sensitivity Analysis of Variable Redundancy in Cost Categories**

To assess the impact of potentially redundant variables, we conducted supplementary analyses by excluding 10 aggregated variables representing total costs across different agricultural processes (transportation, harvesting, spraying, pest management, weed management, water management, fertilizing, growing, seed preparation, soil preparation, and miscellaneous). S1 Table presents the performance comparison of themodels with lowest RMSE values from this modified dataset, while S1 Fig illustrates the SHAP global feature importance distribution for the top-performing model. Our findings indicate minimal impact on predictive performance, with RMSE changes of less than 0.01 units, confirming that the AutoML framework effectively managed potential redundancy in the dataset. Although the removal of aggregated cost variables caused a redistribution of feature importance weights—with rental cost (harvesting) exhibiting elevated influence—the five most influential features maintained their relative ranking positions, confirming the stability of our key insights.

**S1 Table. Results of Sensitivity Analysis - Variable Redundancy in Cost Categories**

| **Model Type** | **RMSE** | **MAE** | **R²** | **MSE** |
| --- | --- | --- | --- | --- |
| **Full feature model** | 0.532 | 0.372 | 0.538 | 0.283 |
| **Without total cost variables** | 0.540 | 0.374 | 0.525 | 0.291 |
| **Performance difference** | 0.008 | 0.002 | 0.013 | 0.008 |
| **Percentage change** | 1.5% | 0.5% | 2.4% | 2.8% |
