# Supplementary material for: Explainable machine learning reveals diverse yield-determining factors among Thai rice farmer cohorts: Implications for targeted agricultural support
Source: PLoS One. 2026 Jun 15;21(6):e0349688. doi: 10.1371/journal.pone.0349688 (PMC13268196; doi:10.1371/journal.pone.0349688)
Supplement: S2 File — (DOCX) [file pone.0349688.s003.docx]

**Automated Machine Learning Frameworks**

**Auto-sklean framework**

Auto-sklearn is an advanced automated machine learning (AutoML) framework that won the first and second international AutoML challenge. At its core, auto-sklearn utilizes meta-learning to warm-start the optimization process, leveraging information from previous experiments on similar datasets to inform initial configurations. The framework then employs Bayesian optimization, specifically the Sequential Model-based Algorithm Configuration (SMAC), to efficiently search through the vast configuration space of machine learning algorithms and their hyperparameters. Auto-sklearn's component library includes an array of classifiers, regressors, feature preprocessors, and data preprocessors, which are automatically selected and combined to construct optimal pipelines. A key feature of auto-sklearn is its ensemble construction mechanism, which builds an ensemble of the best-performing models discovered during the optimization process, using an ensemble selection technique to choose and weight models effectively [42].

Implementation

From our dataset, auto-sklearn (version 0.14.6) was configured to optimize for the mean squared error (MSE) metric, with a time limit of 600 seconds per run time and 14,400 seconds for the task. Resampling strategy was set to “cv” or cross-validation with resampling strategy arguments as 5-fold. The calculation was performed using Python 3.8.

**Autogluon framework**

AutoGluon is an automated machine learning (AutoML) framework that streamlines the development of high-performance machine learning models. It employs a multi-layer stacking approach, automatically training a diverse set of base algorithm models and then combining their predictions using additional models in subsequent layers. The framework begins by preprocessing the input data, handling tasks such as missing value imputation and categorical encoding. It then proceeds to train a variety of models, including neural networks, gradient boosting machines, and traditional algorithms like random forests, adapting its strategy based on the specific dataset and problem type. AutoGluon's strength lies in its multi-layer stack ensembling approach, which combines multiple models through sophisticated weighting schemes and adaptive time-budgeting strategies. The framework employs model-specific hyperparameter optimization and automated feature engineering to maximize predictive performance while efficiently managing computational resources [52]. AutoGluon also provides interpretability features, offering insights into feature importance and model decisions [43].

Implementation

We modified several default parameters of AutoGluon for fair comparison with other frameworks. The included_model_types parameter was set to "XGB", "GBM", "CAT", "XT", "RF", "NN_TORCH", and "FASTAI", representing a diverse array of algorithms as implemented with other frameworks. To ensure fair comparison with other frameworks that have limited runs or time constraints, we set AutoGluon's preset parameter to "good_quality" (the third-highest accuracy setting), as AutoGluon does not offer a direct parameter to limit the number of runs. We enabled auto_stack by setting it to true, activating AutoGluon's advanced multi-layer stacking functionality for ensembling. Finally, num_bag_folds was set to 5, implementing a 5-fold validation. The framework was run using Python 3.8 with Autogluon version v1.0.0.

**h2o framework**

h2o AutoML is an advanced automated machine learning framework that employs a comprehensive approach to model development and optimization. Unlike AutoGluon, which focuses on multi-layer stacking, or auto-sklearn, which primarily relies on Bayesian optimization, h2o utilizes a combination of random grid search and a modified genetic algorithm for hyperparameter tuning. The framework begins by automatically preprocessing the input data and then trains a diverse set of algorithms, including gradient boosting machines, random forests, deep neural networks, and generalized linear models. A key differentiating feature of h2o is its intelligent resource allocation, dynamically adjusting the time spent on different algorithms based on their performance and the overall time budget, which contrasts with the fixed time allocation in auto-sklearn [42, 44]. h2o generates a leaderboard of models, including both individual algorithms and stacked ensembles, providing users with a range of options and insights into model performance [44]. h2o’s stacked ensembles often achieve superior performance compared to individual models, a feature shared with AutoGluon but implemented differently [43]. While all four frameworks chosen for the study offer some level of model interpretability, h2o provides robust features including variable importance analysis and partial dependence plots. Lastly, h2o provides a web-based interface, Flow, an interactive environment for model building and visualization, making it accessible to users with varying levels of programming expertise [44].

Implementation

We implemented the framework using Python 3.8 and h2O v.3.4. The max_models was set to 60 and n-folds was 5 for fair comparison and consistency in cross-validation approach.

**mljar framework**

mljar is an automated machine learning framework that distinguishes itself through its emphasis on transparency, interpretability, and user-friendly reporting. mljar employs a multi-step process that includes extensive exploratory data analysis, feature engineering, and a diverse set of machine learning algorithms. The framework begins with a comprehensive data analysis phase, generating detailed statistical summaries and visualizations. mljar then proceeds with automated feature engineering, including handling of missing values, encoding of categorical variables, and creation of interaction terms. In contrast to h2o's focus on distributed computing for large datasets, mljar is designed to be efficient on single-machine setups, making it accessible to a wider range of users [44-45]. The framework utilizes a combination of random search and a genetic algorithm for hyperparameter optimization, similar to h2o but different from auto-sklearn's Bayesian optimization approach [42]. A key differentiating feature of mljar is its extensive model explanation capabilities, providing detailed insights into feature importance, decision trees, and partial dependence plots for each model. Unlike h2o's leaderboard approach, mljar generates a comprehensive report that includes not only model performance metrics but also detailed explanations and visualizations for each model, facilitating easier understanding [44-45].

Implementation

We implemented mljar in Python 3.8. The mode was set to “Optuna”, leveraging its advanced hyperparameter optimization algorithms, with an extended time budget of 14,400 seconds to allow for thorough exploration of the hyperparameter space. We set the explanation level to 2, enabling model interpretability features, including feature importance and SHAP values. The validation strategy was set to 5-fold cross-validation, with shuffling and stratification to ensure consistent evaluation across folds. We enabled ensemble training and model stacking for fair setting with the other frameworks.
