# Supplementary material for: Explainable machine learning reveals diverse yield-determining factors among Thai rice farmer cohorts: Implications for targeted agricultural support
Source: PLoS One. 2026 Jun 15;21(6):e0349688. doi: 10.1371/journal.pone.0349688 (PMC13268196; doi:10.1371/journal.pone.0349688)
Supplement: S3 File — (DOCX) [file pone.0349688.s004.docx]

**Evaluation Metrics**

**RMSE**

RMSE is a widely used measure in regression tasks, particularly suitable for agricultural yield predictions due to its sensitivity to large errors and its expression in the same units as the target variable [47]. The RMSE serves to aggregate the magnitudes of the errors in predictions into a single measure of predictive power. It is calculated as the square root of the average of squared differences between predicted and observed values:

$$RMSE = \sqrt{((\frac{1}{n})\times\sum{(y_{pred} - y_{true})}^{2}}$$

where $y_{pred}$ represents the predicted rice yield, $y_{true}$ is the observed yield, and n is the number of observations.

**MAE**

Mean Absolute Error (MAE) was employed as one of the model evaluation metrics. MAE measures the average magnitude of errors between predicted and actual values, without considering their direction. It is calculated as:

$$MAE= (\frac{1}{n})\times\sum|y_{pred}-y_{true}|$$

where n is the number of observations, $y_{true}$ represents the actual values, and $y_{pred}$ represents the predicted values.

MAE treats all individual differences equally and expresses the average model prediction error in the original units of the variable being predicted. Unlike RMSE, MAE is less sensitive to outliers and provides a more straightforward interpretation of average model error [47]. Lower MAE values indicate better model performance.

**R^2^**

The coefficient of determination (R²) was used to evaluate the model's goodness of fit. R² measures the proportion of variance in the dependent variable that is predictable from the independent variable(s), ranging from 0 to 1. It is calculated as:

$$R^{2} = 1 - \frac{SS_{res}}{SS_{tot}}$$

where $SS_{res}$ is the sum of squared residuals and $SS_{tot}$ is the total sum of squares.

R² values closer to 1 indicate that a greater proportion of variance is explained by the model, with 1 representing a perfect fit. Conversely, an R² of 0 indicates that the model explains none of the variability in the response variable [48]. While R² provides an intuitive measure of model fit, it should be interpreted alongside other performance metrics as it can be insensitive to proportional and additive differences between predictions and observations.
