# Supplementary material for: Explainable machine learning reveals diverse yield-determining factors among Thai rice farmer cohorts: Implications for targeted agricultural support
Source: PLoS One. 2026 Jun 15;21(6):e0349688. doi: 10.1371/journal.pone.0349688 (PMC13268196; doi:10.1371/journal.pone.0349688)
Supplement: S4 File — (DOCX) [file pone.0349688.s005.docx]

**Model explainability using SHapley Additive exPlanations (SHAP) Values**

**Theoretical Foundation**

SHAP values are derived from Shapley values in cooperative game theory. In the context of machine learning, features are treated as "players" in a cooperative game, where the prediction is the "payout". The SHAP value for a feature represents its marginal contribution to the prediction, averaged over all possible feature coalitions [23].

**Calculation of SHAP Values**

For a given instance, SHAP values are computed as follows:

1. All possible feature subsets are considered.

2. For each subset, the difference in prediction with and without the feature of interest is calculated.

3. These differences are weighted based on the subset size and averaged.

The resulting SHAP value $\phi_{i}$for feature *i* is given by:

$$\phi_{i} = \Sigma\frac{|S|!(|F|-|S|-1)!}{|F|!} [fx(S\cup\{i\}) - fx(S)]$$

Where F is the set of all features, S is a subset of features not including i, and fx is the model prediction function. |F| is the size of set F, or the total number of features available for prediction. |S| is the size of set S or the size of feature subsets not including feature i. This weighs the marginal contribution of i across different combinations.

Implementation

For AutoGluon and auto-sklearn, we used shap.KernelExplainer class from SHAP Python library V 0.45.0. For h2o, we used the function h2o.explain(). For mljar, we set the parameter explain_level to 2.

Note that for AutoGluon, we calculated SHAP values from a sample of 250 farmers from the dataset to reduce its significant computational overhead for KernelExplainer. To estimate SHAP values for a single sample, KernelExplainer calls the inference function twice: first with the sample unaugmented, and then with many randomly augmented instances of the sample. The theoretical justification for this sampling approach lies in the statistical properties of SHAP values. As shown by Covert and Lee [53], SHAP values are expectations over all possible feature orderings, and thus can be effectively estimated using Monte Carlo sampling methods. This sampling approach converges to the true SHAP values as the sample size increases, providing a robust approximation even with a subset of the data. Lundberg and Lee demonstrated that sampling-based approximations of Shapley values can provide reliable feature importance estimates while significantly reducing computational overhead [51].

**Interpretation of SHAP Values**

SHAP values are interpreted as follows:

1. The magnitude of a SHAP value indicates the feature's importance for a particular prediction.

2. The sign of the SHAP value shows whether the feature increased (positive) or decreased (negative) the prediction relative to the baseline.

3. SHAP values sum to the difference between the model's prediction and the expected prediction for the baseline.

**Global and Local Explanations**

We generated both global and local explanations:

1. Global explanations: Aggregate SHAP values across the dataset to understand overall feature importance and model behavior.

2. Local explanations: Compute SHAP values for individual predictions to explain specific model decisions.

**Visualization**

SHAP values were visualized using shap Python library V0.46.0.
