# Supplementary material for: Explainable machine learning reveals diverse yield-determining factors among Thai rice farmer cohorts: Implications for targeted agricultural support
Source: PLoS One. 2026 Jun 15;21(6):e0349688. doi: 10.1371/journal.pone.0349688 (PMC13268196; doi:10.1371/journal.pone.0349688)
Supplement: S5 File — (DOCX) [file pone.0349688.s006.docx]

**Clustering Farmers Based on SHAP Values Using UMAP and DBSCAN**

To identify distinct groups of farmers based on the influence of various factors on their rice yields, we employed a two-step approach combining Uniform Manifold Approximation and Projection (UMAP) for dimensionality reduction and Density-Based Spatial Clustering of Applications with Noise (DBSCAN) for clustering. This method was applied to the SHAP (SHapley Additive exPlanations) values, allowing us to cluster farmers with similar feature importance patterns.

**6.5.1 SHAP Value Calculation**

We obtained instance-level SHAP values for each farmer using the SHAP library [51] applied to our best-performing AutoML model. These SHAP values represent the contribution of each feature to the predicted rice yield for individual farmers.

**6.5.2 Dimensionality Reduction with UMAP**

To address the high-dimensionality of the SHAP value space and improve clustering efficiency, we applied UMAP [54] as a preprocessing step:

1. We used the UMAP implementation from the umap-learn library (version 0.5.6).

2. After trial and error, the final UMAP parameters were set as follows:

- n_neighbors: 13 (balancing local and global structure preservation)

- min_dist: 0 (ensuring cohesion)

- n_components: 2 (best separation for clusters)

3. We applied UMAP to reduce the SHAP value matrix from 54 features to a 2-dimensional representation.

**6.5.3 DBSCAN Clustering**

We then applied DBSCAN [55] to the UMAP-reduced data:

1. We used the DBSCAN implementation from scikit-learn (version 1.0) [70]

2. To determine optimal DBSCAN parameters, we employed a grid search approach:

- eps values ranged from 0.1 to 1.0 (with 0.1 increments)

- min_samples values ranged from 5 to 20

3. We selected the parameter combination that maximized the silhouette score which is eps:0.4 and min_samples:15

**Cluster validation**

To validate the stability and robustness of our clustering results, we:

1. Performed multiple runs of UMAP with different random seeds to ensure consistency in the reduced representation.

2. Conducted a sensitivity analysis by varying UMAP and DBSCAN parameters to assess the stability of cluster assignments.

**Visualization**

We generated the plot using seaborn v0.13.2 [71], and matplotlib v3.8.4 Python library [72].

**References**

70. Pedregosa F, Varoquaux G, Gramfort A, Michel V, Thirion B, Grisel O, et al. Scikit-learn: Machine Learning in Python. J Mach Learn Res. 2011 Nov;12(null):2825–30.

71. Waskom M. seaborn: statistical data visualization. J Open Source Softw. 2021 Apr 6;6(60):3021.

72. Hunter JD. Matplotlib: A 2D graphics environment. Comput Sci Eng. 2007;9(3):90–5.
