# Supplementary material for: Explainable machine learning reveals diverse yield-determining factors among Thai rice farmer cohorts: Implications for targeted agricultural support
Source: PLoS One. 2026 Jun 15;21(6):e0349688. doi: 10.1371/journal.pone.0349688 (PMC13268196; doi:10.1371/journal.pone.0349688)
Supplement: S6 File — (DOCX) [file pone.0349688.s007.docx]

### **Data preparation**

### For machine learning prediction, we used yields as target variable and calculated as yields per hectare by the following calculation

###

$$Yield \left( \frac{tonnes}{hectare} \right)= Yield \left( \frac{tonnes}{rai} \right)x 6.25$$
